# EGB761 ameliorates chronic cerebral hypoperfusion-induced cognitive dysfunction and synaptic plasticity impairment

**DOI:** 10.18632/aging.202555

**Published:** 2021-02-03

**Authors:** Zhao-Hui Yao, Jing Wang, Jing-Ping Yuan, Kai Xiao, Shao-Feng Zhang, Yan-Chun Xie, Jun-Hua Mei

**Affiliations:** 1 Department of Geriatrics, Renmin Hospital of Wuhan University, Wuhan, China; 2 Department of Pathology, Renmin Hospital of Wuhan University, Wuhan, China; 3 Department of Neurology, Renmin Hospital of Wuhan University, Wuhan, China

**Keywords:** chronic cerebral hypoperfusion, EGB761, cognition dysfunction, synaptic plasticity

## Abstract

Chronic cerebral hypoperfusion (CCH) may lead to the cognitive dysfunction, but the underlying mechanisms are unclear. EGB761, extracted from *Ginkgo biloba* and as a phytomedicine widely used in the world, has been showed to have various neuroprotective roles and mechanisms, and therapeutic effects in Alzheimer’s disease and other cognitive dysfunctions. However, improvements in cognitive function after CCH, following treatment with EGB761, have not been ascertained yet. In this study, we used the behavior test, electrophysiology, neurobiochemistry, and immunohistochemistry to investigate the EGB761’s effect on CCH-induced cognitive dysfunction and identify its underlying mechanisms. The results showed that EGB761 ameliorates spatial cognitive dysfunction occurring after CCH. It may also improve impairment of the long-term potentiation, field excitable potential, synaptic transmission, and the transmission synchronization of neural circuit signals between the entorhinal cortex and hippocampal CA1. EGB761 may also reverse the inhibition of neural activity and the degeneration of dendritic spines and synaptic structure after CCH; it also prevents the downregulation of synaptic proteins molecules and pathways related to the formation and stability of dendritic spines structures. EGB761 may inhibit axon demyelination and ameliorate the inhibition of the mTOR signaling pathway after CCH to improve protein synthesis. In conclusion, EGB761 treatment after CCH may improve spatial cognitive function by ameliorating synaptic plasticity impairment, synapse degeneration, and axon demyelination by rectifying the inhibition of the mTOR signaling pathway.

## INTRODUCTION

Chronic cerebral hypoperfusion (CCH) occurs concomitantly with vascular dementia and Alzheimer’s disease (AD) [1, 2]; it not only promotes the progression of AD but also independently causes vascular dementia in some patients [3, 4]. From causing mild cognitive changes to obvious cognitive impairment due to vascular factors, CCH functioned in progress of these neurodegenerative diseases. Owing to CCH, blood supply to the brain areas related to cognitive function is reduced, and the neurons and nerve fibers in these areas experience prolonged durations of nutrient, energy, and oxygen deficits [5]. Glucose hypometabolism leads to oxidative–nitrosative stress (ONS) and neuroinflammation, which disrupts the blood-brain barrier and affects neurons [6], causes white matter lesions that impair the neural circuits [7, 8], decreases the clearance of toxic metabolites [9], reduces synaptic proteomes [10], promotes tau hyperphosphorylation [11], inhibits neurotransmitter synthesis and release [12], impairs synaptic plasticity [13], etc. These mechanisms work together to impair cognitive functions. Nevertheless, existing knowledge of the pathogenesis of CCH is scarce. Therefore, the treatment of CCH-induced cognitive dysfunction has been ineffective and unsatisfactory.

EGB761 is a phytomedicine extracted from *Ginkgo biloba*. Results of animal experiments have shown that EGB761 significantly ameliorated the cognitive deficits of aging db/db (-/-) mice [14]; it also improved cognitive dysfunction in the APP/PS1 mouse through the inhibition of inflammatory responses [15]. Furthermore, EGB761 can protect the blood-brain barrier against Aβ1-42 oligomer-induced damage [16] and oxidative stress [17]. It may also increase the level of neurotransmitters [18], protect mitochondrial function in AD mice, weaken excitotoxicity-induced neuronal death after brain ischemia [19], and improve age-related impairments in synaptic plasticity and excitability [20]. However, Cochrane trails and meta-analysis have shown that the clinical benefits of *Ginkgo biloba* in patients with cognitive impairment are inconsistent [21, 22]. Some studies have shown that EGB761 did not produce the expected therapeutic effect on cognitive impairment [23]. Hence, further studies are required to ascertain whether EGB761 can improve cognitive dysfunction after CCH and investigate the molecular mechanisms underlying the clinical and beneficial effects.

In this study, we investigated the mechanisms that cause cognitive dysfunction after CCH, including impairment in synaptic plasticity, and the molecular basis for the pathology. We also investigated whether EGB761 improved CCH-induced cognitive dysfunction and enhanced synaptic plasticity. The findings will reveal the effects, and the related mechanisms, of EGB761 on cognitive impairment after CCH, which will provide a clear and relatively definite theoretical proof from different angles for the treatment of clinical vascular dementia.

## RESULTS

### EGB761 improves hippocampus-dependent spatial cognition dysfunction in rats after CCH

**Figure 1:**
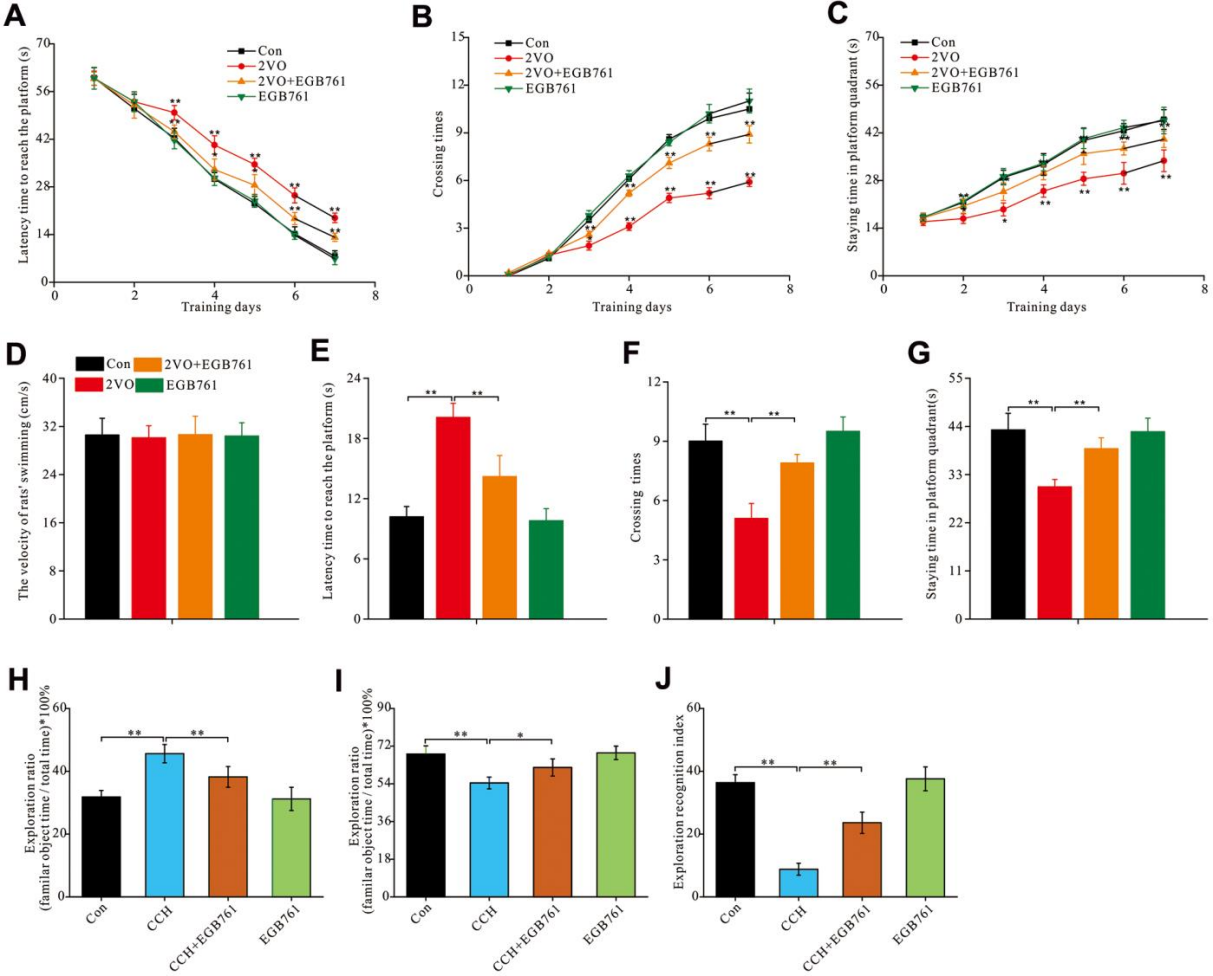
**EGB761 could improve hippocampus-dependent spatial cognition dysfunction after CCH in rats in MWM and NOR test.** The spatial cognitive function were examined by MWM. The latency finding the platform (**A**), crossing times (**B**) and staying time (**C**) in platform areas were recorded and analyzed the learning abilities. The all rats’ swimming velocity was recorded to evaluate the moving abilities (**D**). The short-term memory was assessed by recording and analyzing latency finding the platform (**E**), crossing times (**F**) and staying time in platform areas (**G**) after removing the platform. NOR test was carried out for further evaluated the rats’ spatial cognition. The staying time on familiar and novel object were recorded. Then the exploration ratio (**H**, **I**), and exploration recognition index were counted and analyzed (**J**). Con: the sham group (n=14); 2VO: the group receiving CCH by 2-vessel occlusion (n=12); 2VO+EGB761: the group receiving 2-vessel occlusion and EGB761 treatment (n=14); EGB761: the sham group receiving EGB761 treatment (n=13). *, *P*<0.05; **, *P*<0.01.

To investigate whether EGB761 could improve cognitive dysfunction after CCH, the hippocampus-dependent spatial cognitive ability was investigated by MWM. During the 7-day training, the 2VO group rats showed the longer escape latency to reach the platform than the control group (*P*< 0.01, from the 3^rd^ to 7^th^ training day), but 2VO+EGB761 group had significantly shorter escape latency than 2VO group (*P* < 0.01, on the 3^rd^, 6^th^, and 7^th^ day; *P* < 0.05, on the 4^th^ and 5^th^ day) (Figure 1A). 2VO group had fewer platforms crossing times and shorter time spent on the platform quadrant than control group (*P* < 0.01, on the 3^rd^ to 7^th^ day). 2VO+EGB761 group took shorter the duration of latency to reach the platform compared to 2VO group (*P*<0.01, from 3^rd^ to 7^th^ day; *P* < 0.05, 2^nd^ and 3^rd^ day; *P* < 0.01, from 4^th^ to 7^th^ day) (Figure 1B, 1C). Moreover, there was no difference between the swimming velocities of the experimental rats (Figure 1D). After a day of rest, following platform removal, 2VO group had the longer latency finding platform than control group (*P* < 0.01); But 2VO+EGB761 group spent shorter latency than 2VO group (*P* < 0.01) (Figure 1E). Moreover, 2VO group had the less platform crossing times and the shorter staying time than control group, but 2VO+EGB761 group had improved these than 2VO group (*P* < 0.01) (Figure 1F, 1G).

To further validate the cognitive improvements after CCH following EGB761 treatment, the NOR test was used to examine the spatial cognitive function. For NOR test, 2VO group had the higher ratio of familiar objects exploration than control group and 2VO+EGB761 group (*P* < 0.01) (Figure 1H). Accordingly, the 2VO group had less exploration ratio of novel objects than control group, but 2VO+EGB761 had higher exploration ratio than 2VO group (*P* < 0.01) (Figure 1I). Finally, 2VO group had the lower exploration recognition index (ERI) than control group, but 2VO+EGB group had the higher ERI than 2VO group (*P* < 0.01) (Figure 1J).

These findings meant that CCH induced spatial cognitive dysfunction, and that EGB761 treatment could prevent this dysfunction.

### EGB761 could improve LTP impairment, synaptic transmission dysfunction, and the synchronization of neural circuit signals between the entorhinal cortex and hippocampal CA1 after CCH

**Figure 2:**
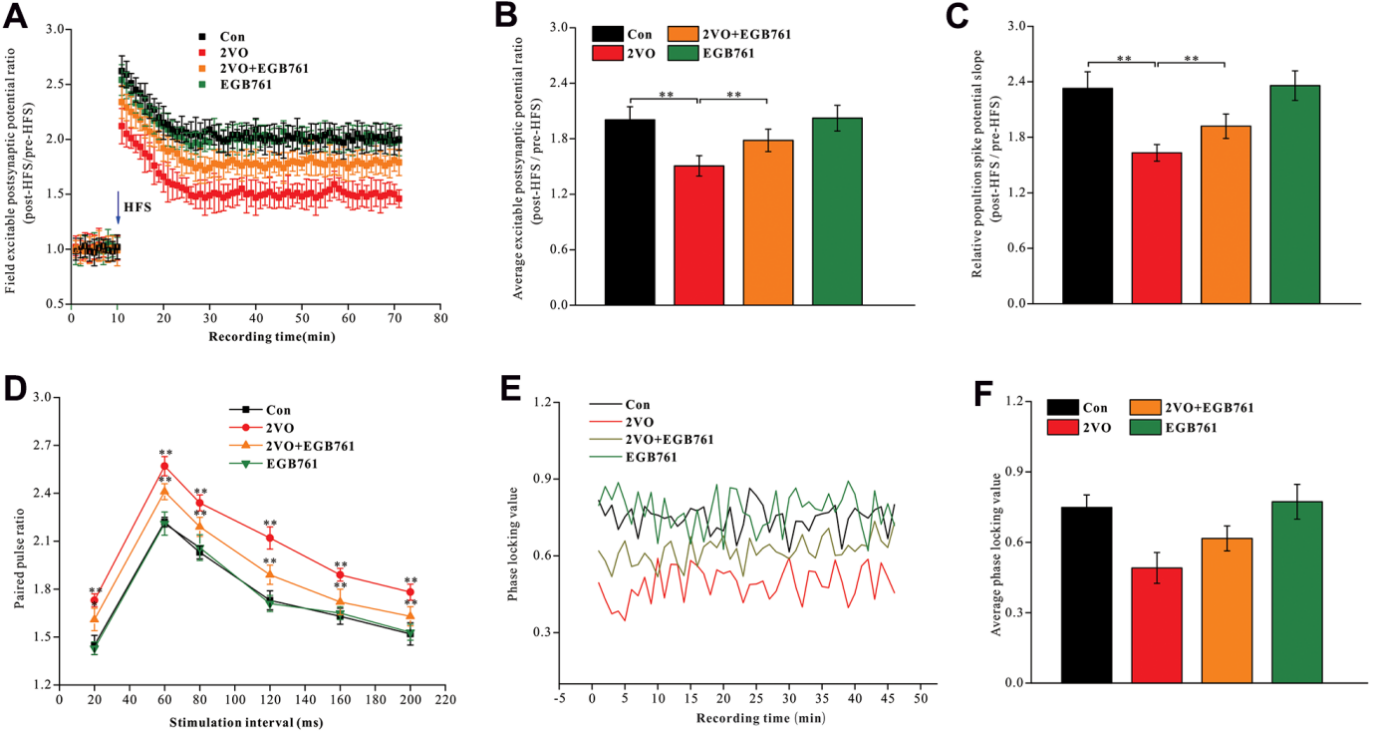
**EGB761 could improve LTP impairment, synaptic transmission dysfunction and the synchronization of neural circuit signals between the entorhinal cortex and CA1 of hippocampus after CCH.** After finishing behavior tests, the stimulating and recording electrodes were implanted in entorhinal cortex and CA1 of hippocampus. The rats received the HFS in entorhinal cortex and the excitable postsynaptic potentials during pre-HFS and post-HFS were recording and analyzed (**A**, **B**). The average population spikes potential were recorded and the potential slopes were counted (**C**). The paired pulse potential (20ms, 60ms, 80ms, 120ms, 160, 200ms time interval) were recorded (**D**). The electrical signals in cortex and CA1 of hippocampus were recorded and phase locking values were calculated (**E**) and analyzed (**F**).

Synaptic function plasticity, indicated by LTP, is a key mechanism underlying cognition. To investigate the mechanisms underlying cognitive dysfunction after CCH, the excitable postsynaptic field potentials in the hippocampal CA1 were recorded pre- and post-HFS of the entorhinal cortex to observe LTP (Figure 2A). 2VO group had the significantly lower average EPSP ratios and the population spike potential (pre-HFS over post-HFS) (*P* < 0.01), but 2VO+EGB group had higher average EPSP ratios and the population spike potential compared to 2VO group (*P* < 0.01) (Figure 2B, 2C).

PPF reflects the presynaptic signal transmission ability of the GABAergic interneurons. As PPF increases, the presynaptic and trans-synaptic transmission abilities of neural signal decrease [48, 49]. The trisynaptic neural circuit between the hippocampal CA1 and the entorhinal cortex was closely related to cognition. We observed that 2VO group had a higher PPF during stimulation intervals of 20, 60, 80, 120, 160, and 200 ms (*P* < 0.01), whereas 2VO+EGB761 group had lower PPF than 2VO group (*P* < 0.01) (Figure 2D).

The neural circuit synchronization between the entorhinal cortex and CA1 was closely related to the cognitive function. The neural signals were simultaneously recorded in CA1 and the entorhinal cortex and they were transformed with the Hilbert transform function. The phase of neural signals was extracted, and the locking phase values were calculated (Figure 2E). The data showed that 2VO group had significantly lower PLV (*P* < 0.01), but 2VO+EGB761 group had higher PLV than 2VO group (*P* < 0.01) (Figure 2F).

These results meant that CCH induced LTP impairment, synaptic transmission dysfunction, and the desynchronization of neural circuit signals between the entorhinal cortex and hippocampal CA1, and that EGB761 treatment could prevent them.

### EGB761 and CCH did not change the neuron density and apoptosis in the hippocampus

**Figure 3:**
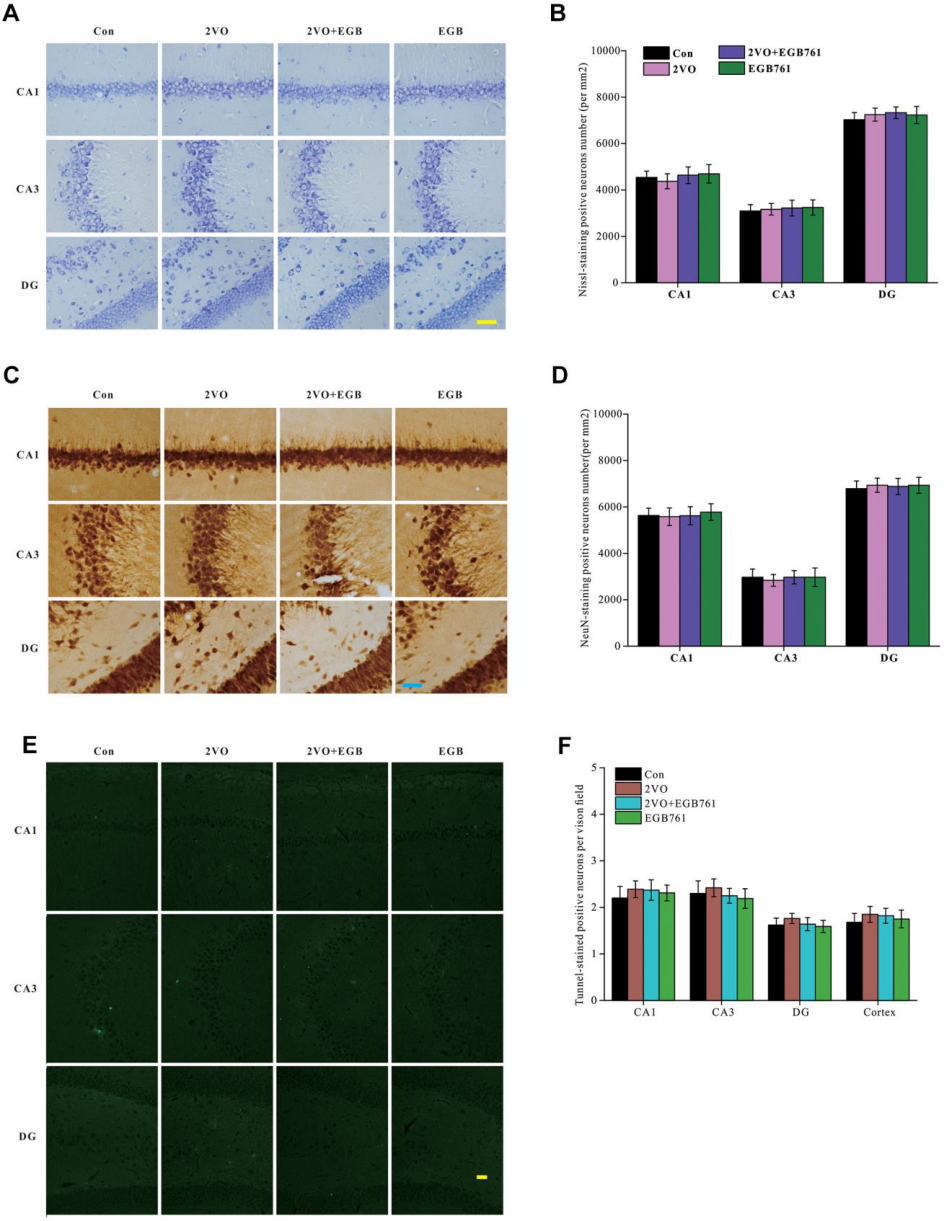
**CCH did not change the neurons density and apoptosis in hippocampus**. The brain sections were stained by Nissl staining solutions, NeuN antibody and TUNNEL staining kit. Nissl staining slides were showed (**A**) (Bar scale= 25μm) and neurons in subregions of hippocampus were counted (**B**). NeuN labeled neurons were showed (**C**) (Bar scale= 25μm) and neurons in subregions of hippocampus were counted (**D**). TUNNEL staining section was showed (**E**) (Bar scale= 100μm) and counted (**F**) (n=3 for 4 group).

To identify the mechanisms underlying cognitive dysfunction occurring after CCH, the brain slices (5 μm) were stained with Nissl solution and anti-NeuN antibody (30-μm slice) to detect the number of alterations within the hippocampal neurons. Images of Nissl-stained and anti-NeuN-labeled neurons showed that there are no differences for the neuronal densities in the different sub-regions of the hippocampus among the control group, 2VO and 2VO+EGB761 groups (*P* > 0.01) (Figure 3A, 3D). These observations suggested that CCH and EGB761 did not change the neuronal number of hippocampus. Although the neuronal number remained unchanged, it was possible that the neurons were about to die. To determine whether neuronal apoptosis due to CCH worsened cognition, TUNEL staining was performed to detect neuronal apoptosis. The dUDP-stained neurons showed no significant aggravation in apoptosis (Figure 3E, 3F), which suggested that the hippocampal neurons did not undergo apoptosis after CCH, and that cognitive impairment could be functional or structural.

### EGB761 could relieve the inhibition of neural activity and the structural degeneration after CCH

**Figure 4:**
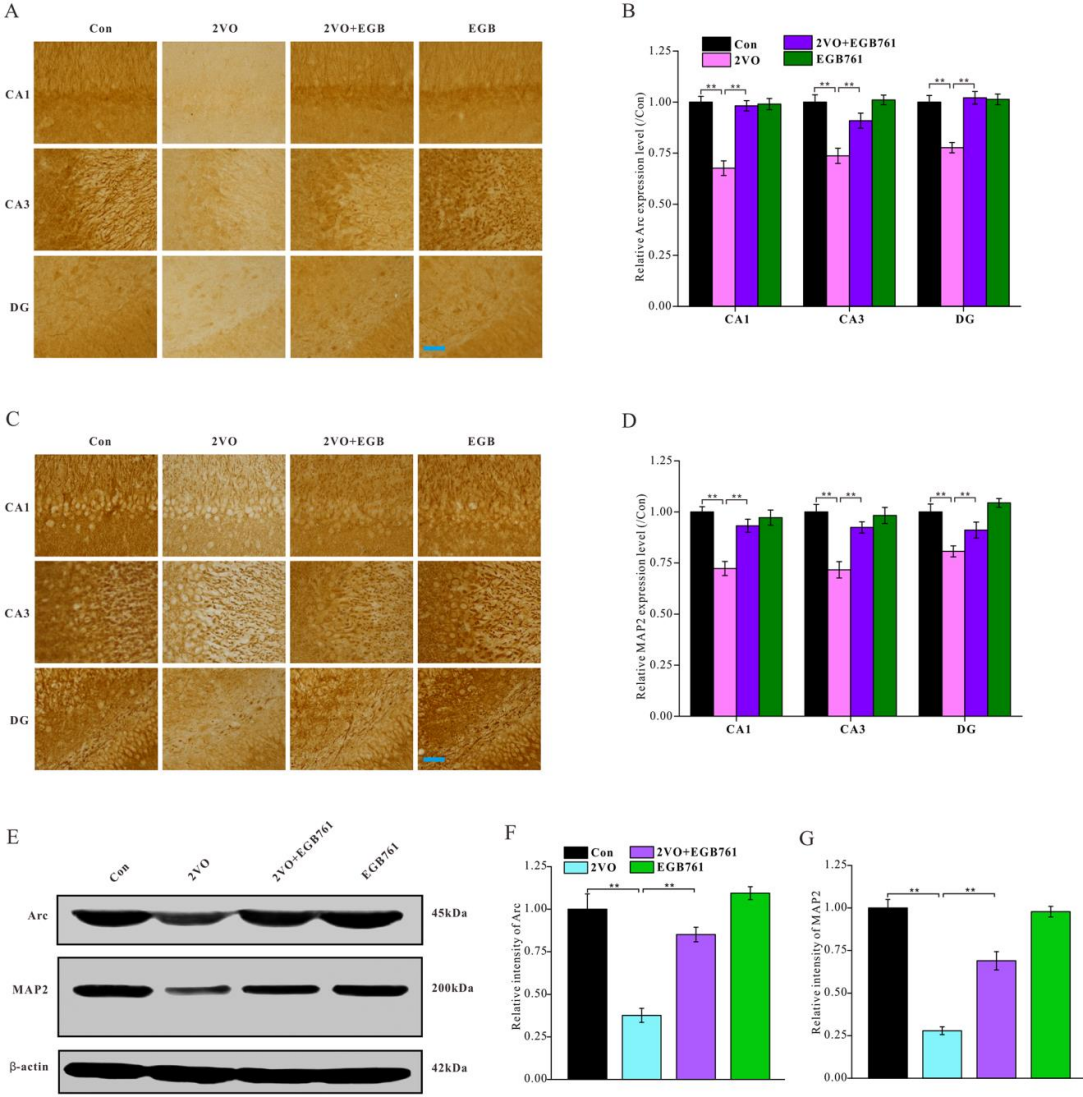
**EGB761 could relieve the inhibition of neural activity and the structural degeneration of neurons after CCH.** The brain sections were developed by Arc (**A**) and MAP2 (**C**) antibody to evaluate the neural activity and the structural degeneration. The Arc and MAP2 particles were observed and analyzed in subregions of hippocampus (**B**, **D**) (n=3 for 4 group). The hippocampus homogenate was relatively quantitatively assayed by Western blotting with Arc and MAP2 antibody (**E**, **F**, **G**). (Con: n=4; 2VO: n=5; 2VO+EGB761: n=6; EGB761: n=5).

Neuronal activity is important for cognition. To investigate whether the respective cognitive effects of 2VO and EGB761 involves impairment of neuronal activity, the Arc protein, a neuronal activity marker, was investigated. Immunohistochemical staining for Arc protein showed that 2VO group had the significantly lower Arc level (*P* < 0.01), but 2VO+EGB761 group had higher Arc level that 2VO group (*P* < 0.01) (Figure 4A, 4B). Western blotting also showed that likely Arc level as immunohistochemical staining (*P* < 0.01) (Figure 4E, 4F). These results meant the cognitive dysfunction occurring after CCH involved impairment of neuronal activity, and that EGB761 could improve it.

To investigate whether structural degeneration of neurons occurred after CCH, microtubule associated protein 2 (MAP2), a marker of neuronal structural degeneration, was investigated. Both immunohistochemical staining and western blotting showed 2VO group had the lower MAP2 level (*P* < 0.01), but 2VO+EGB761 group had higher MAP2 level than 2VO group (*P* < 0.01). This finding suggested CCH induced the structural degeneration of neurons, and that EGB761 could prevent it (Figure 4C–4F).

### EGB761 could prevent the CCH-induced degeneration of dendritic spines and the downregulation of molecules and pathways related to the formation and stability of dendritic spines

**Figure 5:**
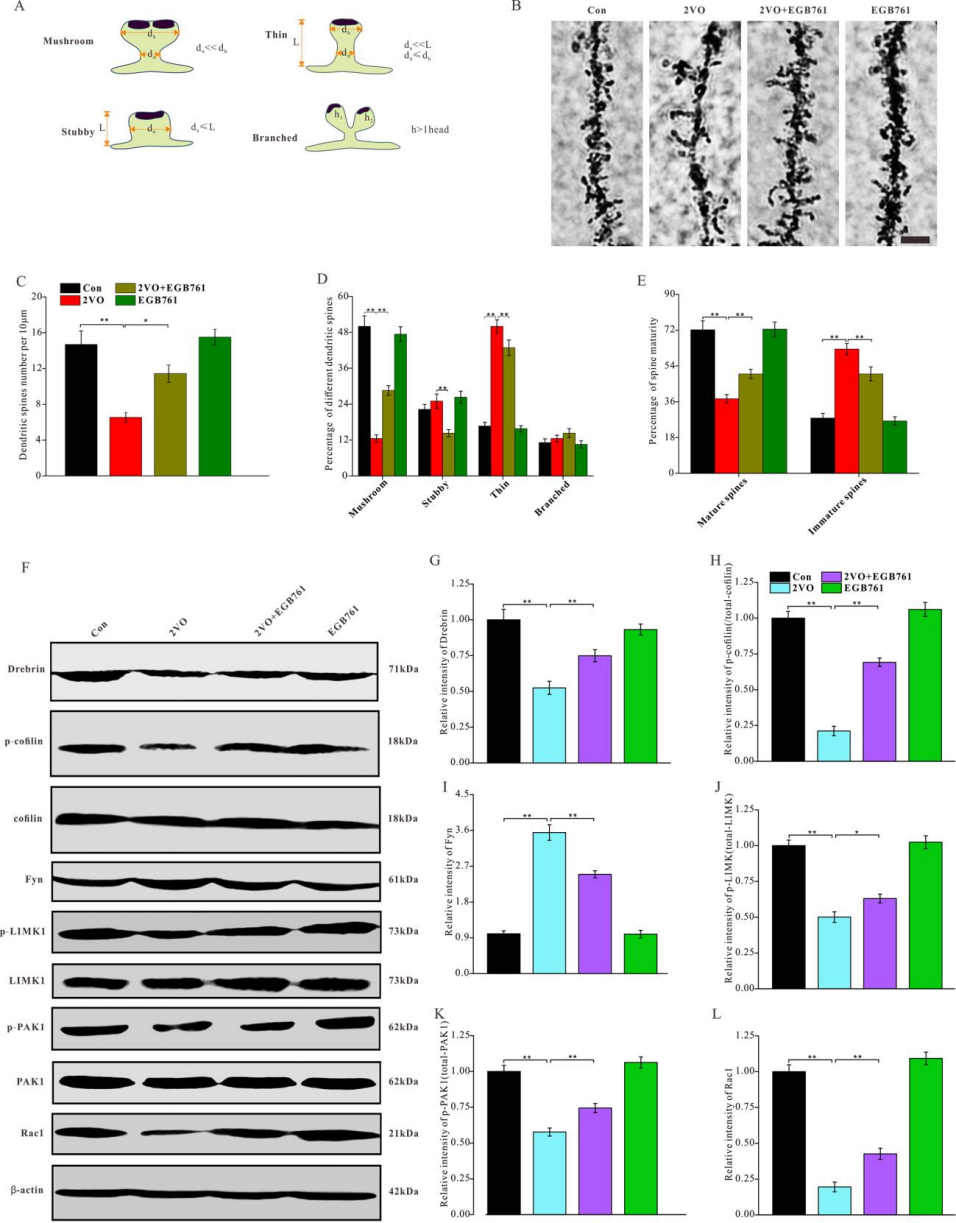
**EGB761 could prevent the degeneration of dendritic spines and downregulation of molecules and pathways related to the formation and stability of dendritic spines after CCH**. The rats’ brains were fixed with formaldehyde and impregnated with Golgi staining solution to observe the dendritic spines. (**A**) Schematic diagram of morphological classification of dendritic spines was showed. (**B**) The dendritic spine were observed in 100μm sections (Bar scale=5μm) and density (**C**), percentage of different kinds of spines (**D**), and maturity (**E**) of dendritic spines were counted and calculated (n=3 for 4 group). The brain homogenates were investigated and relatively quantitatively analyzed by proteins blotting for Drebrin, p-cofilin, t-cofilin, Fyn, p-LIMK1, t-LIMK1, p-PAK1, t-PAK1, Rac1 and β-actin antibody (**F**–**L**).

The structural plasticity of neurons is also pivotal to cognition. In this study, the dynamic alteration of four types of dendritic spines was critical to the structural plasticity of neurons (Figure 5A). To investigate the role of structural plasticity in EGB761-mediated reversal of cognitive dysfunction, the Golgi bodies of the hippocampal tissues were stained to observe and quantify the morphological changes occurring in dendritic spines (Figure 5B). 2VO group had the significantly lower dendritic spine density (per 10 μm) (*P* < 0.01), but 2VO+EGB group had higher spine density than 2VO group (*P* < 0.01) (Figure 5C). 2VO group had the fewer mushroom spines and more thin spines (*P* < 0.01), but 2VO+EGB group had more mushroom spines and less thin spines than 2VO group (Figure 5D). 2VO group had the fewer mature spines and more immature spines, but 2VO+EGB761 group had more mature spines and fewer immature spines than 2VO group (*P* < 0.01) (Figure 5E).

Drebrin can bind with and dissociate from fibrous actin (F-actin) remodel their morphology of dendritic spines. The phosphorylation of cofilin can stabilize F-actin to enhance the stability of dendritic spines formation. The phosphorylation of cofilin is commonly regulated by the Rac1/PAK1/LIMK1 pathway and Fyn protein. Therefore, to investigate the degeneration of dendritic spines after CCH and after EGB761 administration, drebrin, cofilin; the upstream kinases Fyn, LIMK1, PAK1 and Rac1 were investigated (Figure 5F). The western blotting data showed that 2VO group had the lower level of drebrin and the phosphorylated cofilin, whereas 2VO+EGB761 group had their higher expression of drebrin and the phosphorylated cofilin over 2VO group (*P* < 0.01) (Figure 5G, 5H). This finding suggested that the downregulation of drebrin and phosphorylated cofilin may participate the degeneration of dendritic spines after CCH, but EGB761 treatment may prevent this downregulation to ameliorate the degeneration of dendritic spines occurring after CCH. Moreover, 2VO group had the significantly downregulated p-LIMK1, p-PAK1, and Rac1 proteins than control group, but 2VO+EGB761 group had the higher levels of these proteins over 2VO group (*P* < 0.01) (Figure 5J–5L). However, we found that 2VO group had the upregulated kinase Fyn (*P* < 0.01), and 2VO+EGB761 group had less Fyn level over 2VO group (*P*<0.01) (Figure 5I). Together, these data meant that cofilin phosphorylation by Rac1/PAK1/LIMK1 pathway may take part in dendritic spines degeneration, but EGB761 treatment may have upregulated cofilin phosphorylation by the Rac1/PAK1/LIMK1 pathway to ameliorate the degeneration of dendritic spines.

### EGB761 could ameliorate the degeneration of synaptic structures and upregulated the synaptic proteins

**Figure 6:**
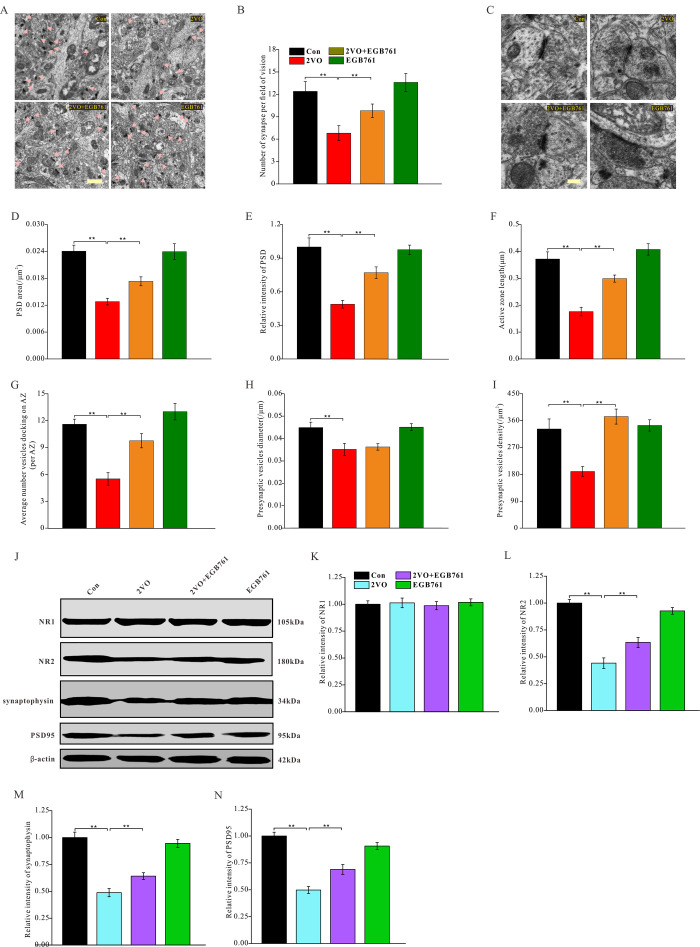
**EGB761 could increase synapse density, improve degeneration of synapse structure and prevent downregulation of synaptic proteins after CCH**. The hippocampi tissues were made into ultramicrotomed sections with negative staining. The synapse was observed (**A**) (Bar scale= 1μm) and synapse density per vision fiend was counted (**B**). The structure of synapse was observed (**C**) (Bar scale= 250nm) and PSD area (**D**), relative PSD density (**E**), active zone length (**F**), average number of vesicles docking on AZ (**G**), presynaptic vesicles diameter (**H**), and presynaptic vesicle density (**I**) (n=3 for 4 group). NR1, NR2, synaptophysin, PSD95, and β-actin of the brain homogenates were examined by proteins blot and assayed (**J**–**N**).

Keeping synaptic structure normal is very critical for the cognition-related neural circuit formations. To investigate the alterations in the synaptic structure of the hippocampal CA1 neurons after 2VO and EGB761 treatment, ultrathin sections of the CA1 were negatively stained and observed under a TEM (Figure 6A, 6C). The TEM imaging showed that 2VO group had the markedly reduced synaptic density than control group, but 2VO+EGB761 group had more synaptic density than control group (*P* < 0.01) (Figure 6B). Moreover, in 2VO group, the PSD area, relative PSD intensity, active zone length, and the average number of vesicles docking on the active zone were lower, but in 2VO+EGB761 group these microstructure were improved over 2VO group (*P* < 0.01) (Figure 6D–6G). Interestingly, 2VO group had the less vesicle diameters and the presynaptic vesicle densities over control group (*P* < 0.01). However, 2VO+EGB761 group showed the more presynaptic vesicle density over 2VO group (*P* < 0.01) (Figure 6H–6I). These observations meant CCH could induce synaptic degeneration, whereas EGB761 could prevent it.

Synaptic proteins are the fundamental components of synaptic structures. To identify the mechanisms underlying synaptic structure changes, several synaptic proteins of hippocampus were investigated after CCH (Figure 6J). The data demonstrated that 2VO group had noticeably downregulated NR2, synaptophysin, and PSD95, whereas 2VO+EGB761 group had more these synaptic proteins than 2VO group (*P*<0.01) (Figure 6L–6N). However, in all the groups, no change was observed in the expression levels of NR1 (Figure 6K). These findings suggested that CCH could induce the downregulation of synaptic proteins to deteriorate synaptic structures, but that EGB761 could prevent this downregulation to ameliorate the degeneration of synaptic structures.

### EGB761 could inhibit CCH-induced axon demyelination

**Figure 7:**
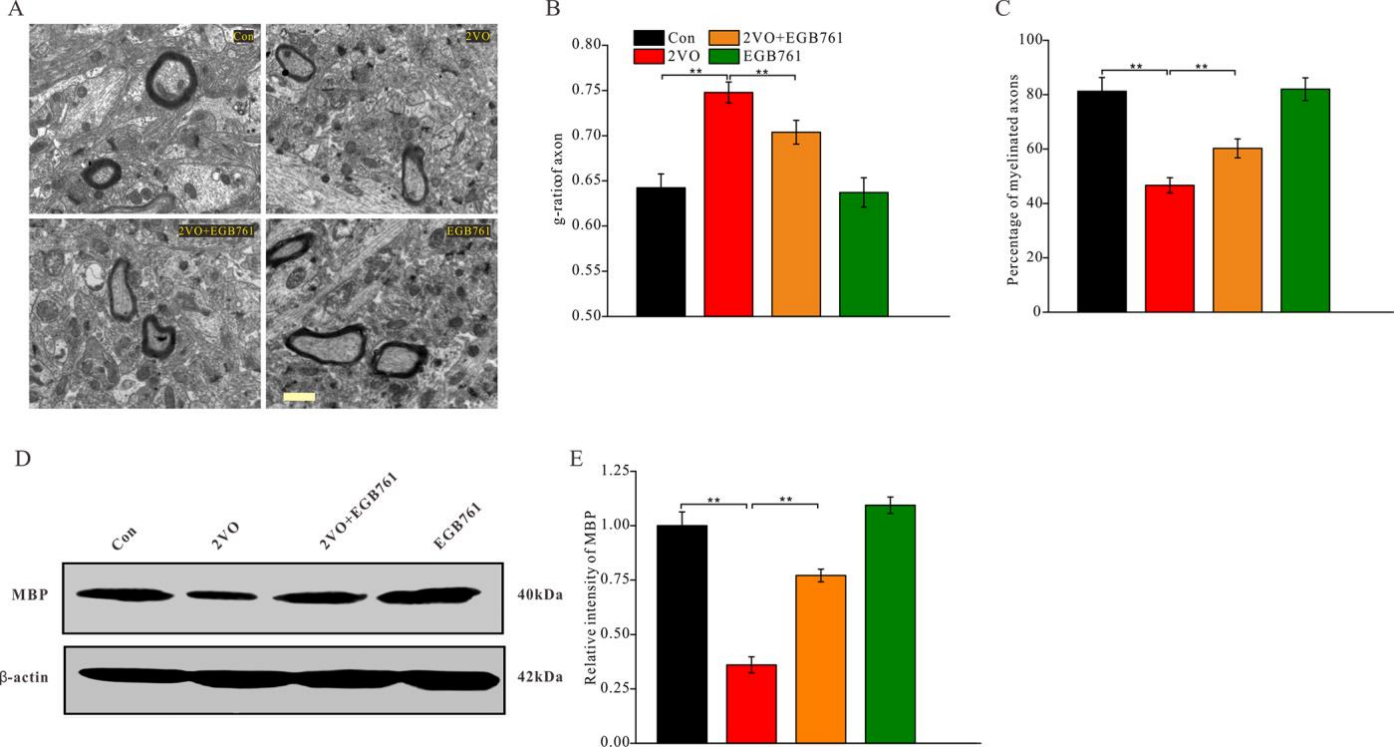
**EGB761 could inhibit axons demyelination after CCH**. Under the ultramicrotomed sections, the axons and their myelination was observed (**A**) (Bar scale= 1μm) and diameter of axonal fiber and total diameter of axon (axonal fiber myelin sheath) were measured. g-ratio (diameter of axonal fiber / total diameter of axon) and percentage of myelinated axons were calculated (n=3 for 4 group) (**B**, **C**). MBP and β-actin in brain homogenates were assayed by Western blotting (**D**, **E**) (Con: n=4; 2VO: n=5; 2VO+EGB761: n=6; EGB761: n=5).

Axon demyelination might impair cognition by reducing the transmission velocity of neural signals. The alterations in axon myelination after CCH were investigated with a TEM (Figure 7A). The axon myelination analysis showed that 2VO group had the higher g-ratio, but 2VO+EGB761 group demonstrated lower g-ratio than 2VO group (*P* < 0.01) (Figure 7B). The lower percentage of myelinated axons in 2VO group was less over control group, but 2VO+EGB761 group exhibited higher percentage of myelinated axons than 2VO group (*P* < 0.01) (Figure 7C). These implied that CCH induced axon demyelination, and that EGB761 treatment could prevent it. Myelin basic protein (MBP) is the main constituent protein of the myelin sheath of CNS. To further validate axon demyelination, MBP protein level was detected with western blotting (Figure 7D). 2VO group had less MBP protein level than control group, but 2VO+EGB761 group had more MBP protein than 2VO group (*P* < 0.01) (Figure 7E). These findings further clarified that EGB761 could prevent CCH-induced axon demyelination.

### EGB761 could prevent CCH-induced against mTOR signaling pathway inhibition

**Figure 8:**
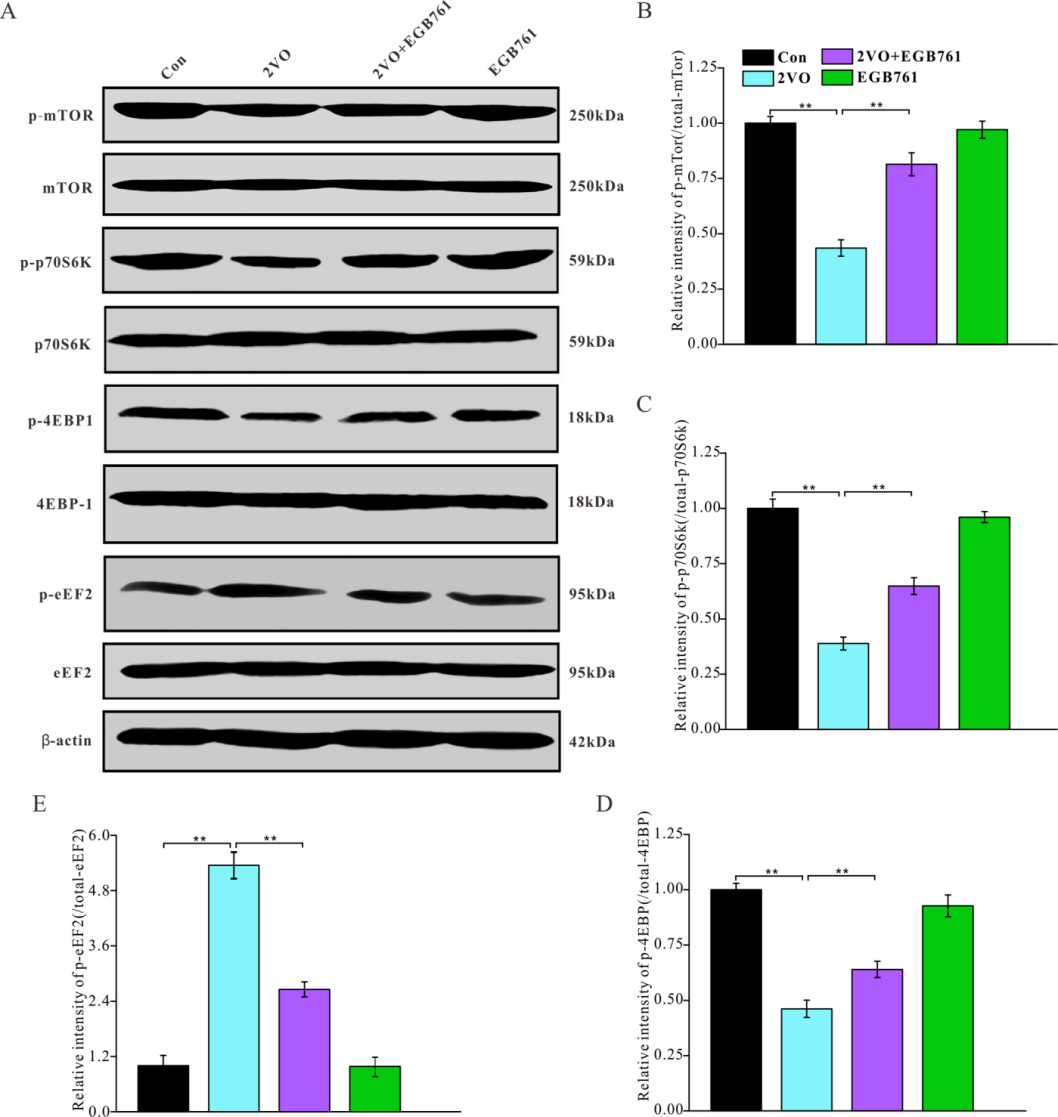
**EGB761 could improve inhibition of mTor signaling pathway after CCH.** p-mTor, t-mTor, p-p70S6K, p70S6K, p-4EBP1, 4EBP1, p-eEF2, eEF2, and β-actin in brain homogenates were assayed by Western blotting (**A**–**E**) (Con: n=4; 2VO: n=5; 2VO+EGB761: n=6; EGB761: n=5).

2VO made several proteins decrease, including Arc, c-Fos, NR2, synaptophysin, PSD95, drebrin, and MBP. For regulating protein synthesis, the mTOR signaling pathway plays the very important role. To investigate the mechanisms regulating the downregulation of these proteins, the expression of mTOR signaling pathway molecules were detected with western blotting (Figure 8A). 2VO group had less p-mTOR, p-p70S6K and p-4EBP1 levels, but 2VO+EGB761 group manifested the higher levels of these proteins than 2VO group (*P* < 0.01) (Figure 8B–8D). 2VO group showed higher p-eEF2 level, but 2VO+EGB761 group had lower p-eEF2 level than 2VO group (*P* < 0.01) (Figure 8E). The activated mTOR could phosphorylate p70S6K, and the activated p-p70S6K could further phosphorylate 4EBP1. The phosphorylated 4EBP1 dissociates from the eukaryotic translation initiation factor 4E, which then facilitates protein translation. Hence, it can be speculated that CCH could induce the inhibition of protein synthesis, and that EGB761 treatment could reverse this inhibition.

## DISCUSSION

In this study, we found that treatment with EGB761 may improve CCH-induced spatial cognitive dysfunction. EGB761 may also improve LTP impairment, synaptic transmission, and the synchronization of neural circuit signals between the entorhinal cortex and hippocampal CA1, as well as relieve the inhibition of neural activity and the degeneration of dendritic spines and synapse occurring after CCH. Furthermore, EGB761 may prevent CCH-induced downregulation of proteins and pathways related to the formation and stability of dendritic spines and synaptic proteins as well as inhibit axon demyelination. Finally, we found that EGB761 can reverse the inhibition of the mTOR signaling pathway occurring after CCH to favor proteins synthesis. These findings suggested that EGB761 may improve spatial cognitive dysfunction by ameliorating the synaptic plasticity impairment, synaptic degeneration, and axon demyelination by rectifying the inhibition of the mTOR signaling pathway occurring after CCH.

During the progress of the cognition-related neurodegenerative diseases, including vascular dementia and AD, CCH played the noticeably important roles. Because of the lack of energy and blood supply to the brain after CCH, normal cognition is affected and impaired. A previous study reported that EGB761, a herbal extract from *Ginkgo biloba*, has good effects against various cognition deficits in elderly db/db (-/-) diabetic mouse, APP/PS1 mouse, and rats with hyperhomocysteinemia [14, 15, 24]. EGB761 also reportedly improves inherited and acquired cognitive impairments. Our study showed that EGB761 could significantly improve spatial cognitive dysfunction occurring after CCH. What were the mechanisms underlying the effect of EGB761 on cognitive dysfunction after CCH? The neuronal apoptosis and reduction in neuronal number may lead to cognition and other neurological deficits [25]. Nevertheless, in this study, no obvious change of neurons number was found, which means that spatial cognitive dysfunction was not induced by an alteration in neuronal numbers. Synaptic plasticity is critical to normal cognition, and an impairment of synaptic plasticity is followed by cognitive deficits [26]. LTP is indicative of synaptic plasticity, and it was used to investigate the cognitive dysfunction occurring after CCH in this study; our results showed reduced LTP after CCH, which suggested that cognitive dysfunction after CCH may be induced by the impaired synaptic plasticity. Our present findings showed that the synaptic transmission and synchronization of neural circuit signals between the entorhinal cortex and hippocampal CA1 were impaired after CCH, and that EGB761 treatment could ameliorate these impairments. The maintenance of synaptic transmission and the synchronization of cognition-related neural circuit signals are important for cognition [27]. EGB761 could prevent and repair the CCH-induced dysfunction of neural signal transmission and synchronization to improve spatial cognitive deficits.

Our present study demonstrated that CCH could induce a decreased proportion of mushroom dendritic spines and mature spines; however, EGB761 could prevent this decrease. Synaptic plasticity includes functional plasticity and structural plasticity. The structural plasticity of synapses is mainly manifested by the dynamic changes in the morphological state of dendritic spines [28]. Generally, the dendritic spines have four morphological states: mushroom, stubby, thin, and branched spines (Figure 5A). The mushroom and stubby forms, which are the mature dendritic spines, can form stable and functional synapses with other spines; however, the thin and branched dendritic spines cannot because they are immature [29]. Cognitive dysfunction occurs when the dendritic spines of hippocampus degenerate [30]. This observation suggests that EGB761 may attenuate CCH-induced spatial cognitive dysfunction by preventing the deterioration of synaptic structural plasticity. Our present findings showed that CCH induced the downregulation of drebrin and the dephosphorylation of cofilin, with corresponding changes in their upstream regulator pathway, while EGB761 treatment prevented these effects. The polymerization of F-actin regulates the shape of dendritic spines. Drebrin in dendritic spines, an actin-binding protein, can function in maintaining the shape of the spine by regulating the formation of stable F-actin. When drebrin level decreases, the dendritic spines may shrink, deform, and even degenerate [31]. Cofilin is another actin-binding protein; when cofilin is activated by dephosphorylation, the cofilin-actin filaments are stably bundled; this sequestration of cofilin by actin causes synaptic dysfunction, damages the normal actin dynamics, and blocking axonal or dendritic transportation [32]. Therefore, these results suggest that EGB761 may increase the expression of drebrin protein and partially inactivate cofilin to prevent dendritic spine degeneration after CCH.

Synapses are the basic connections of neural circuits; they are also the adapters for communication between neurons [33]. Synapses help to obtain learning- and memory-related information from outside the body and transmit them to the brain for processing, integration, and storage, which manifests as cognitive functions [34]. The present study showed that CCH induced synaptic loss, decreased the level of synaptic proteins, and caused synapse degeneration—characterized by a smaller PSD area and lower intensity, shorter active zone length, fewer vesicles docking on the active zone, and shorter presynaptic vesicle diameters. PSD and presynaptic active zone are important functional areas of the synapse, comprising several synaptic proteins [35]. The vesicles docking on the active zone determine the number of vesicles to be released, while the presynaptic vesicle diameter determine the amount of neurotransmitters to be released after each stimulation [36]. All these factors work together to maintain normal synaptic function. EGB761 may prevent CCH-induced synaptic loss, reduction of synaptic proteins, and ameliorate synapse degeneration; these findings provide evidence to state that EGB761 can effectively improve the cognitive dysfunction occurring after CCH.

In this study, the expression of several proteins was downregulated, such as Arc, NR2, and PSD95. We also found that the phosphorylation of mTOR, p70S6K, and 4EBP1 decreased after CCH, and that EGB761 could prevent these effects. One of the most important functions of mTOR, a serine-threonine kinase, is to phosphorylate and activate p70S6K to inhibit protein synthesis. The activated p70S6K can further phosphorylate 4EBP1, an important protein synthesis inhibitor. 4EBP1 can inhibit its assembly into the eIF4F complex to repress translation initiation through binding with the eukaryotic translation initiation factor 4E (EIF4E) [37]. It has been suggested that EGB761 could prevent the inhibition of 4EBP1 by maintaining the mTOR signaling pathway to sustain translation initiation, thereby mitigating the effect of CCH. In our study, we also found that the phosphorylation of eukaryotic elongation factor 2 (eEF2) increased after CCH, and that EGB761 may prevent its phosphorylation-mediated inhibition. eEF2 is an essential factor for promoting the elongation process of protein synthesis, and the phosphorylation-mediated inactivation of eEF2 inhibits protein translation [38]. Hence, it was postulated that EGB761 may protect against CCH-mediated translation inhibition by preventing eEF2 phosphorylation to foster translation elongation. In addition, the mTOR signaling pathway can regulate autophagy, which can also contribute to cognitive dysfunction [39]. Whether CCH causes autophagy and whether EGB761 can inhibit autophagy will be investigated in a future study.

We found that EGB761 could improve cognitive dysfunction by protecting neural synaptic plasticity and by upregulating some synaptic proteins and axonal constituent proteins; however, it remains to be conclusively verified whether these molecular mechanisms played important roles in cognitive dysfunction after CCH and its reversal with EGB761 treatment. These aspects would be investigated in future studies by regulating the expression of the altered synaptic proteins to verify their roles.

In conclusion, we found that EGB761 may improve spatial cognitive dysfunction, rectify synaptic transmission, and promote synchronization of neural signals in neural circuits. EGB761 may also relieve the degeneration of dendritic spines, synapses, and axon myelination and ameliorate the inhibition of the protein synthesis pathway. EGB761 has shown potential for improving cognitive dysfunction caused by CCH, but several potential mechanisms, such as inhibiting autophagy, counteracting oxidative stress, promoting cerebral metabolisms, etc., need to be investigated and clarified further.

## MATERIALS AND METHODS

### Antibodies and chemicals

All primary antibody and secondary antibody in the present study were listed in Table 1. The BCA protein assay kit was from Pierce Chemical Company (Rockford, IL, USA). Diaminobenzidine (DAB) chromogenic kit, biotin-labeled secondary antibodies, and horseradish peroxidase-labeled antibodies was purchased (Zhongshan Goldenbridge Biotechnology Co., Ltd). *In Situ* Cell Death Detection Kit was ordered (Roche, Inc., Roche, Germany). EGB761 was from Dr. Willmar Schwabe Pharmaceuticals (KG, Karlsruhe, Germany).

**Table 1:** All antibodies used in this study were showed.

**Primary antibodies**					
**Anti-Target**	**Abbreviation**	**Host**	**Company and source**	**Catalog number**	**Application**
activity-regulated cytoskeleton-associated protein/activity-regulated gene 3.1	Arc/Arg 3.1	Rabbit	Proteintech Wuhan, China	16290-1-AP	WB(1:1000), IHC(1:200)
c-Fos	c-Fos	Mouse	Servicebio Wuhan, China	GB12069	WB(1:1000), IHC(1:200)
NeuN	NeuN	Rabbit	Servicebio Wuhan, China	GB11138	IF(1:200)
microtubule-associated protein 2	MAP2	Rabbit	Proteintech Wuhan, China	17490-1-AP	WB(1:1000), IHC(1:200)
Developmentally-regulated brain protein	drebrin	Rabbit	Servicebio Wuhan, China	GB111519	WB(1:500)
phopsphorylated cofilin	p-cofilin	Rabbit	Cell signaling Beverly, MA, USA	3313	WB(1:1000)
total cofilin	t-cofilin	Rabbit	Cell signaling Beverly, MA, USA	5175	WB(1:1000)
phopsphorylated LIM kinases1	p-LIMK1	Rabbit	abcam Cambridge, CB, UK	ab194798	WB(1:1000)
total LIM kinases1	t-LIMK1	Rabbit	Cell signaling Beverly, MA, USA	3842	WB(1:1000)
Fyn	Fyn	Rabbit	abcam Cambridge, CB, UK	ab125016	WB(1:1000)
phopsphorylated p21-activated kinase1	p-PAK1	Rabbit	abcam Cambridge, CB, UK	ab75599	WB(1:1000)
total p21-activated kinase1	t-PAK1	Rabbit	Cell signaling Beverly, MA, USA	2602	WB(1:1000)
total Rac1	Rac1	Rabbit	Cell signaling Beverly, MA, USA	4651	WB(1:1000)
N-methyl D-aspartate receptor 1	NR1	Rabbit	Proteintech Wuhan, China	27676-1-AP	WB(1:1000)
N-methyl D-aspartate receptor 2	NR2A/2B	Rabbit	Millipore Billerica, MA, USA	ab65783	WB(1:1000)
synaptophysin	synaptophysin	Rabbit	Servicebio Wuhan, China	GB11553	WB(1:1000)
Postsynaptic Density protein 95	PSD95	Mouse	Cell signaling Beverly, MA, USA	36233	WB(1:1000)
myelin basic protein	MBP	Rabbit	Servicebio Wuhan, China	GB11226	WB(1:1000)
phosphorylated mTor	p-mTor	Rabbit	Cell signaling Beverly, MA, USA	5536	WB(1:1000)
total mTor	t-mTor	Rabbit	Cell signaling Beverly, MA, USA	2983	WB(1:1000)
phosphorylated p70S6K	p-p70S6K	Rabbit	Cell signaling Beverly, MA, USA	9204	WB(1:1000)
total p70S6K	t-p70S6K	Rabbit	Cell signaling Beverly, MA, USA	2708	WB(1:1000)
phosphorylated 4EBP-1	p-4EBP-1	Rabbit	Cell signaling Beverly, MA, USA	9459	WB(1:1000)
total 4EBP-1	t-4EBP-1	Rabbit	Cell signaling Beverly, MA, USA	9452	WB(1:1000)
phosphorylated eEF2	p-eEF2	Rabbit	Cell signaling Beverly, MA, USA	2331	WB(1:1000)
total eEF2	t-eEF2	Rabbit	Cell signaling Beverly, MA, USA	2332	WB(1:1000)
β-actin	β-actin	Mouse	Cell signaling Beverly, MA, USA	3700	WB(1:1000)
**Secondary antibodies**					
Anti- Target	Host	Label	Company	Catalog number	Application
Rabbit IgG	Goat	Biotin	Zsbio Beijing, China	SAP-9101	IHC(1:100)
Mouse IgG	Goat	Biotin	Zsbio Beijing, China	SP-9102	IHC(1:100)
Rabbit IgG	Goat	IRDye™ (800CW)	Licor Lincoln, NE, USA	AB_2651127	WB(1:10000)
Mouse IgG	Goat	IRDye™ (800CW)	Licor Lincoln, NE, USA	AB_2687825	WB(1:10000)
Rabbit IgG	Goat	Alexa Fluor 488	Cell signaling Beverly, MA, USA	4412	IF(1:1000)
Mouse IgG	Goat	Alexa Fluor 488	Cell signaling Beverly, MA, USA	4408	IF(1:1000)

### Animals and chronic cerebral hypoperfusion (CCH) model

80 male adult Sprague-Dawley rats (180-210g) were ordered from the Hunan SJA laboratory animal CO., LTD. The rats had accessible food and water ad libitum, and were housed in the comfortable living environment. All performed animal experiments were reviewed, discussed and approved by Ethics Committee of people's Hospital of Wuhan University.

According to random principle, all rats were assigned to four groups: Control sham group (Con) (n=15), the bilateral common carotid arteries was separated but not ligated, and received saline (10ml/kg weight). The chronic cerebral hypoperfusion rats (2VO) (n=25) had bilateral common carotid arteries ligation and was treated with the saline. 2VO+EGB761 group (n=25), had the 2VO operation and together got EGB761 treatment at the same time for 1month. EGB761 group (n=15), had the EB761 treatment for 1month but no 2VO operation.

The rats were injected intraperitoneally with chloral hydrate (0.4g/kg) to be anesthetizated. Both common carotid arteries were separated, freed and ligated [40, 41]. In control sham rats, the common carotid arteries separate surgery was made while the vessels were not ligated. The operated rats with the less than 70 per cent cerebral blood flow by a laser Doppler system were as to be the qualified CCH rats [41].

### Drug treatment

EGB761 tablets were dissolved in saline, remove impurities by filtration. The EGB761 solution was prepared at concentration of 10mg/ml. After the 2VO operation, the rats were injected intraperitoneally EGB761 solution (100mg/kg weight) for 1 month [42, 43].

### Morris water maze

After 1 month CCH or EGB761 treatment, all rats were trained and acquired the short-term spatial memory with the Morris water maze (MWM). Detailed MWM method was as previous [41]. The rats were trained 3 times from different quadrant to remember an underwater invisible platform during 7 consecutive days. The rats’ swimming tracks to look for the platform were recorded [44]. Trajectory data were analyzed to obtain the information of crossing times around the platform areas, the staying time in different areas, and the latency to find the platform. After 1-day rest, the rats were re-tested for detect the memory retention.

### Novel object recognition test (NOR test)

Rodents instinctively explored the novel objects, so that, by taking advantage of this feature, the NOR test was introduced to evaluate cognitive dysfunction [29]. NOR test was our previous study [45]. On 1^st^ day, the rats were familiar with a the open-field consisting of 55cm×55cm×38cm plexiglass box and two objects. On 2^nd^ day, after replacing the previous objects with two similar novel objects, the rats explored the objects for 5min. On 3^rd^ day, after replaced one of objects with a different one, the rats explored the objects for 5min to get the memory retention on two different objects. Time ratio of exploring the two objects and the exploration discrimination index was calculated [46].

### Electrophysiology

Electrophysiological recording was also as previous study [28]. After finishing the spatial cognitive investigation, the rats were anaesthetized by intraperitoneal injection of urethane (1.6 g/kg, i.p.). The recording electrode and stimulating electrode were implanted according to the coordinates of rat brain atlas (CA1 and entorhinal cortex). Field excitatory postsynaptic potential (fEPSPs) between CA1 region and entorhinal cortex was recorded with the 3 kHz sampling frequency. The high frequency stimulation was imposed. LTP was calculated with the ratio of post- and pre- high frequency stimulation (HFS). The paired pulse facilitation (PPF) can show presynaptic activity [47]. Briefly, 60% of max fEPSP responses were given for two stimuli. Two stimuli interval ranged from 20ms to 60ms, 80ms, 120ms, 160ms and 200ms was employed to introduce PPF. The ratio of the two interval fEPSP value was calculated to decide the PPF.

### Neural signal data acquisition and PLV calculation

Cerebral electrical signals were recorded with neural recording data acquisition system (from Thinker Tech Nanjing Biotech Co., Ltd.), sampled at 500Hz and stored for offline analysis with Matlab 7.0 software.

The phase locking value (PLV) can be used to investigate the synchronization of the narrowband electrical brain activities signals [48]. During low frequency band, PLV is calculated by the average instantaneous phase difference [49]. To determine whether the spikes synchronization in entorhinal cortex and CA1 in theta waves, theta phases of LFPs in the two brain areas were acquired through calculating the angle of the Hilbert transform of the theta of spikes. Then PLV was calculated as following:

$$\text{PLV}\;=\;\frac{1}{N}|\;\sum_{i=1}^{N}\left(\exp\;\left(\text{j}\;\;*\;\;{\left(\varphi ENT\;-\;\varphi CA1\right)}_{i}\right)\right)\;|$$

(φENT-φCA1)_i_ is the theta phase difference between the entorhinal cortex(ENT) and CA1 at time i. PLV ranges from 0 to1, and as the PLV value increases, the phase locking of the signal increases.

### Immunohistochemistry and immunofluorescence

The rats were perfused 0.9% NaCl and 4% paraformaldehyde in turn. Post-fixed rat brains were embedded and sectioned (5 μm thickness slide). After 30-minutes antigen retrieval, the slides were developed with primary antibodies for 12 hours at 4° C (1:200). For immunohistochemistry, the slides were developed with biotin-labeled secondary antibodies and horseradish peroxidase-labeled antibodies. Diaminobenzidine (DAB) was used to react for final staining. After dehydrated and transparent, the slides were mounted and observed under an Olympus BX60 microscope (Tokyo, Japan). For immunofluorescence, after incubation with primary antibody, the slides were developed with the FITC-labeled secondary antibodies.

### Nissl staining

The brain slices were immerged in cresyl violet solution for Nissl staining under 37° C for 20min and separated with 70% alcohol solution. The images were observed under the microscope. Nissl-stained neurons of the hippocampus were counted and analyzed by Image-Pro software 6.0.

### Terminal deoxynucleotidyl transferase (TdT)-mediated dUTP nick end labelling (TUNEL)

The rat brain slices were dewaxed, dehydrated and antigen retrieval. The slices were incubated with the TUNEL Kit for 60 min at 37° C as the suggestions of the instruction manual. The TUNEL-positive neurons were observed and analyzed under Olympus microscope [50, 51].

### Golgi staining

The detailed method was as previous our study [44]. Briefly, the rats were perfused, fixed and pre-impregnated with Golgi stain. The rats’ brains were isolated, sectioned cut into 5-mm -thickness tissues and 3-days post-impregnated with Golgi stain. After finishing impregnation, the tissues were sectioned into 100μm slices and stained with 3% silver nitrate staining solution in dark environment. The slices were observed under oil microscope with magnification of 100 times. Dendritic spines were counted, sorted and statistically analyzed.

### Western blot

The hippocampi of rats were isolated and homogenized with tissue lysate mixture consisting the cock tail proteinase inhibitor and phosphatase inhibitor on the ice to avoid degradation. After mixing with 4×loading, boiling denaturation and sonication, BCA method was employed to determine total protein concentration. The total proteins run and differentiated in 10% SDS-PAGE. Then the proteins in gel were transferred, blocked and developed at 4° C with primary antibody for 12 hours. The membrane was developed with IRDye™-label secondary antibody (1:10000) for 2 h at room temperature and to be obtained the proteins bands with Licor infrared imaging instrument (Lincoln, NE, USA). The bands were analyzed over the intensity of β-actin.

### Transmission electron microscopy (TEM)

1mm-thickness hippocampi pieces were fixed with phosphate buffer containing 2.5% glutaraldehyde and post-fixed with phosphate buffer containing 1% osmium tetroxide. After dehydration and embedding, the 70 nm-thickness slides were cut from hippocampal pieces. Next, the slides were negative stained and observed under HT7700 TEM (Hitachi, Japan) [52].

The synapses per vision field were counted to evaluate the synapse density. To investigate the synapse degeneration after CCH, the PSD area, relative intensity of PSD, active zone length, average vesicles docking on active zone, presynaptic vesicles diameter and vesicles density were observed and analyzed. The myelinated axons were counted and the percentage of myelinated axons was calculated. The diameter of axons and their fibre bundles were measured. The g-ratio (the diameter of fibre bundle of axon versus axon diameter) was calculated to evaluate the demyelination of axons [53].

### Statistics analysis

All data are showed as means ± standard error of mean (SEM). They were analyzed with Statistical Product and Service Solutions (SPSS) 22.0 statistical software (Chicago, Illinois, USA). The differences between groups were determined by repeated measurement analysis of variance. The differences of the means were determined by one-way analysis and Dunnett's t test. *P*<0.05 was as the significantly statistical difference for different groups.

### Data availability statement

The data that support the findings of this study are available from the corresponding author upon reasonable request.
